# The Developmental Assessment of Social Communication Ability (DASCA): initial creation and psychometric description

**DOI:** 10.1186/s13229-025-00683-z

**Published:** 2025-10-16

**Authors:** Aaron J. Kaat, Audrey Thurm, Cristan Farmer, Shuting Zheng, Sheila Ghods, Stelios Georgiades, Stephen Kanne, Shrikanth Narayanan, Somer L. Bishop

**Affiliations:** 1 Feinberg School of Medicine, Northwestern University, Chicago, IL USA; 2 National Institute of Mental Health, Bethesda, MD USA; 3 University of Texas, Austin, TX USA; 4 Weill Institute for Neurosciences, University of California, San Francisco, San Francisco, CA USA; 5 Offord Centre for Child Studies, McMaster University, Hamilton, ON Canada; 6 Center for Autism and the Developing Brain, Weill Cornell Medicine, White Plains, NY USA; 7 Viterbi School of Engineering, University of Southern California, Los Angeles, CA USA

**Keywords:** Social communication, Item response theory, Psychometrics, Neurodevelopmental disorders, Autism spectrum disorder

## Abstract

**Objective:**

The dearth of tools to quantify and track growth in social communication ability has been a barrier to understanding and monitoring treatment outcomes for neurodevelopmental disorders. We undertook a multi-staged, multisite study to create the Developmental Assessment of Social Communication Ability (DASCA), a new measure explicitly developed as a clinical outcome assessment for monitoring change—both over the course of development and in response to treatment.

**Methods:**

The DASCA is a caregiver-report instrument created using a mixed-methods approach. Qualitative components of this approach included focus groups and cognitive debriefing interviews. Quantitative components included dimensionality analysis, differential item functioning, and item response theory modeling. The item bank was iteratively refined to assess social communication skills that are typically acquired by early- to middle- childhood.

**Results:**

The final DASCA item bank contains 184 items. Expressive language was a major factor in determining the appropriateness of some items for certain groups of children. Negligible differential item functioning, primarily by age, was observed for some items. However, impact analyses determined that this differential item functioning did not meaningfully impact overall scores.

**Limitations:**

Given that sample size limitations prevented us from using separate samples for exploratory and confirmatory phases of modeling, it will be important to gather additional validity evidence in independent samples, especially as the current data were collected during the COVID-19 pandemic.

**Conclusion:**

The DASCA holds promise as an outcome measure for assessing changes in social communication ability. Ongoing development efforts include creating a computer adaptive test administration to allow for serial assessments using different item sets to yield a consistent score that is sensitive to change.

**Supplementary Information:**

The online version contains supplementary material available at 10.1186/s13229-025-00683-z.

## Background

We have previously defined social communication ability as “the appropriate use and modulation of verbal and nonverbal behaviors during interactions with others” (p.555) [1]. Basic social communication skills, like using eye contact to regulate social interaction, or intentionally directing facial expressions to communicate affect to a social partner, start developing in infancy [2]. These skills develop in conjunction with motor, cognitive, language, and play skills, as children engage in increasingly complex interactions with different social partners across different contexts. Although developmental scientists have long been interested in understanding the typical development of social communication behaviors like joint attention and their foundational importance for language and cognition [3–5], in clinical psychology and psychiatry, social communication is most often discussed in diagnostic terms related to autism spectrum disorder (referred to here as autism). Thus, social communication is often viewed and measured in binary terms (i.e., presence vs. absence of deficit), rather than as a spectrum of abilities that develop over time.

Because of the focus on evaluating *deficits*, available measures of social communication are not responsive indices of severity. Measures such as the Autism Diagnostic Observation Schedule 2nd Edition (ADOS-2 [6]) or the Autism Diagnostic Interview-Revised (ADI-R [7]) prioritize diagnostically salient behaviors, aiming to maximally discriminate between autism and typical development, rather than ensuring measurement coverage at all levels of social communication ability. As a result, meaningful comparisons of social communication ability within or between diagnostic groups are difficult, as is the measurement of within-person change. This has notable implications for some corners of the neurodevelopmental disorder (NDD) field, such as rare genetic diseases, where advances in treatment technology have outpaced our ability to measure their potential effectiveness.

We recently argued that to surmount this challenge, social communication abilities must be disentangled from autism diagnostic criteria and measured within a developmental framework [1, 8]. Such a measure should: (1) respect the developmental nature of social communication and its intersections with language ability and other aspects of development, (2) operationalize indicators of social communication ability as skills rather than deficits, and (3) be sufficiently responsive to change to be used in monitoring or treatment contexts.

To this end, we created a caregiver-report measure of social communication ability, building upon a developmental, skill-based framework and previous psychometric work [9–13]. In the conceptual framework, social communication behavior exhibits a developmental construct shift as children gain more cognitive and language abilities [8, 14]. This is consistent with existing autism diagnostic measures that include different Modules (or item sets) for particular age/language groups in order to focus only on those social-communication behaviors that are most developmentally relevant [6, 15–18]. Our approach followed best practice guidelines for instrument development [19–23], including qualitative work with stakeholders and quantitative modeling. In this report, we describe the multi-phase creation of the Developmental Assessment of Social Communication Ability (DASCA) and provide initial validity evidence in both non-clinical and clinical populations.

## Methods

The methods used to develop the DASCA item bank included both qualitative and quantitative methodology, which can be roughly categorized into initial item pool generation, elicitation of qualitative feedback, and quantitative psychometric evaluation. Importantly, these activities were often co-occurring, iterative, and not sequential. Further, many of these iterative steps occurred during the COVID-19 pandemic, necessitating adaptations for data collection.

### Initial item pool development

We began by developing a conceptual framework for social communication described by Bishop at el [8]. In measurement science, a conceptual framework is a way to connect the theoretical understanding of a construct to the specific behaviors that should be assessed. Our conceptual framework was informed by a literature review, a review of extant measures of social communication, and the expertise of our research team. The conceptual framework [8] facilitated a theory-driven item development process. We initially conceptualized social communication as a multidimensional construct with distinct but related groupings, or types, of social communication behaviors. The conceptual model included behaviors to initiate or terminate interactions, respond to social partners, and actively maintain or extend social engagement. Items varied as to whether they involved basic or foundational social communication skills versus more complex aspects of social cognition, emotion regulation, or behavioral inhibition.

During item creation, we drew upon findings from several previous [9, 10] and new analyses of autism symptom measures, as well as the Vineland Adaptive Behavior Scales (VABS) [24] to inform our focus on ability rather than deficits. Rather than identifying diagnostically discriminating behaviors important for screening and diagnosis, we were most interested in behaviors that were less clearly confounded with age and expressive language ability, and therefore might be more sensitive to change in children already identified as having clinically-significant social communication deficits. Thus, we prioritized social communication behaviors that exhibited variability even among individuals of similar age, expressive language ability, and diagnosis.

Version 1.0 of the candidate bank included 89 *de novo* items. The wording of each item was carefully reviewed to avoid overlap with items from existing measures, as well as to ensure that reading level requirements did not exceed 8th grade, with a preference for below 6th grade. As we developed new items for each version of the candidate item bank, we assigned them by consensus to the relevant parts of the conceptual framework (e.g., initiation, response, maintenance, and/or behavioral regulation; basic social communication skills vs. skills required to navigate increasingly complex social interactions). This was a bidirectionally iterative process; first, we used this information to ensure adequate content coverage [25], adding to aspects of the construct that seemed to have too few relevant items. This allowed us to empirically test whether historical conceptualizations of social communication deficits (e.g., initiations vs. responses; mode of communication vs. pragmatic purpose) were useful for our model. Ultimately, we moved away from conceptualizing social communication as being comprised of separable components (e.g., behaviors involved in initiation, response, maintenance, etc.), because such categories did not reliably emerge from quantitative analyses as described below, and because “success” in social communication requires adequately modulating all of these behaviors. Through the iterative process, we also removed items that required caregiver inferences about a child’s internal state, as well as items that referred to a context outside of the home, as caregivers reported having limited opportunities to observe them.

### Routing items

During item pool development, we considered both the planned computer adaptive format of the final measure and logistical constraints on evaluating a very large item pool. Therefore, we set out to identify caregiver-reported behaviors that might serve as routing items on the DASCA. Using item-level data from the VABS (based on parent-report) together with concurrently collected information about which ADOS-2 Module was selected (based on clinician judgment of language level), we identified specific VABS items that were most associated with the ability to produce more complex language during direct observation [26, 27]. These items were then cross-referenced with language milestones from the Survey of Wellbeing of Young Children [28]. This resulted in four items (put 2 or more words together; use words like “because” or “but”; use prepositions; put verbs in past tense) that were administered as routing items to determine whether a child should receive DASCA questions requiring more or less expressive language.

### Focus groups

The development of the initial item bank was carried out within our immediate research group, but external evaluation was key to its refinement. Thus, we conducted focus groups, first with professionals involved in diagnostic assessment of NDDs, and then with parents/caregivers of individuals diagnosed with NDDs.

The goal of the professional focus groups was to elicit opinions about social communication domain coverage, which was used to edit, add, or eliminate items from the initial item bank. Then, between November 2019 and January 2020, caregiver focus groups reviewed subsets of items from the second major version of the candidate item bank. Caregivers were recruited from an institutional review board (IRB) approved registry of cases clinically referred for a comprehensive developmental evaluation. Of the 11 caregiver participants, four (36%) had children under 5 years and three (27%) had children over 18 years. Six (55%) had children who spoke with phrase speech or better. Participants were divided into caregivers of children who were under 4 years old or with minimal verbal abilities, and caregivers of children who were over 4 years and speaking in phrases or sentences; the groups reviewed items requiring less and more language, respectively. Each group reviewed survey instructions, response options, and 30–40 question stems.

We also conducted cognitive debriefing interviews about the response process for each item included in the item bank [29, 30]. Thirty parents were recruited from clinics affiliated with the authors’ institutions or from the community. Respondents were parents of children or minimally verbal adults with (*n* = 21) or without (*n* = 9) an NDD diagnosis, with a range of racial or ethnic identities (*n* = 19 White, *n* = 6 Asian, *n* = 3 Native American, *n* = 1 African American, *n* = 1 Other). Respondents were asked whether the items were clearly stated, what they were thinking about when they chose a response option, and whether they had ever noticed any changes in their child’s use of the skill(s) captured in each item. They were also invited to provide suggestions about social communication behaviors that they felt were important but not adequately represented in the items they reviewed.

### Field testing

We next conducted mixed methods research to refine and evaluate the item bank. To prevent respondent fatigue, participants completed subsets of items in a planned missingness design [31]. We first fielded the candidate items among parents or caregivers of children and adolescents unselected for any developmental concerns, using a market research firm (Opinions4Good [Op4G]^1^) that maintains a panel of households representing various demographic groups. Respondents in three waves of data collection completed subsets of items from Versions 1–3, as well as free-response items seeking qualitative feedback about the items. A reduced item set (Version 3.1) was then tested in parents/caregivers of children and adolescents with autism, and an unrelated group of siblings of children with autism, all of whom were enrolled in the Simons Powering Autism Research for Knowledge (SPARK) registry. Version 3.1 was also tested in parents/caregivers of children and adolescents with genetic conditions associated with autism and NDDs from the Simons Searchlight registry. The versions and samples for each wave of field testing are summarized in Table 1.

**Table 1:** Participant-related info by method

Data Source	DASCA Version	Sample Size	Dates of collection	Age (months) Mean (SD)	Sex	Race/Ethnicity	Administration Notes
Market Research Firm (Op4G)	Version 1.0	1000	2/12/19 − 3/15/19	51.7 (36.2)	Male: 569 (56.9%) Female: 431 (43.1%) Not Specified: 0 (0%)	Hispanic 238 (23.8%) Non-Hispanic White 589 (58.9%) Non-Hispanic Black 77 (7.7%) Non-Hispanic AI/AN 6 (0.6%) Non-Hispanic Asian 40 (4.0%) Non-Hispanic NHOPI 3 (0.3%) Non-Hispanic Multi-Racial 47 (4.7%)	Anonymous online survey collected between February and March of 2019. Caregivers reported any NDD for *n* = 196 (19.6%), not selected for NDD.
Market Research Firm (Op4G)	Version 2.0	1000	8/31/19 − 9/19/19	77.2 (57.4)	Male: 514 (51.4%) Female: 486 (48.6%) Not Specified: 0 (0%)	Hispanic 134 (13.4%) Non-Hispanic White 676 (67.6%) Non-Hispanic Black 67 (6.7%) Non-Hispanic AI/AN 3 (0.3%) Non-Hispanic Asian 52 (5.2%) Non-Hispanic NHOPI 1 (0.1%) Non-Hispanic Multi-Racial 67 (6.7%)	Anonymous online survey collected September of 2019. Caregivers reported any NDD for *n* = 206 (20.6%), not selected for NDD.
Market Research Firm (Op4G)	Version 3.0	1150	4/7/20 − 4/22/20 and 7/16/20 − 7/29/20	54.4 (50.1)	Male: 576 (50.1%) Female: 574 (49.9%) Not Specified: 1 (0.09%)	Hispanic 191 (16.6%) Non-Hispanic White 733 (63.7%) Non-Hispanic Black 80 (7.0%) Non-Hispanic AI/AN 16 (1.4%) Non-Hispanic Asian 62 (5.4%) Non-Hispanic NHOPI 7 (0.6%) Non-Hispanic Multi-Racial 61 (5.3%)	Anonymous online survey collected in April (*n* = 1000) and July (*n* = 150) of 2020. Caregivers reported any NDD for *n* = 284 (24.7%), not selected for NDD.
Simons Powering Autism Research (SPARK) Research Match—Probands	Version 3.1	1087	6/9/21 − 6/23/21	113.9 (48.0)	Male: 835 (76.8%) Female: 246 (22.6%) Missing/Not Specified: 6 (0.6%)	Hispanic 191 (17.6%) Non-Hispanic White 722 (66.4%) Non-Hispanic Black 41 (3.8%) Non-Hispanic AI/AN 1 (0.1%) Non-Hispanic Asian 28 (2.6%) Non-Hispanic NHOPI 1 (0.1%) Non-Hispanic Multi-Racial 84 (7.7%) Non-Hispanic Other 12 (1.1%) Missing/Not Reported 7 (0.6%)	SPARK is an autism research registry with participants with autism across all ages, and their family members across the U.S. All probands have an NDD by definition. Among siblings, parents reported an NDD for *n* = 346 (33.6%).
SPARK— Siblings	Version 3.1	1184	9/1/21 − 10/15/21	132.2 (50.4)	Male: 580 (49.0%) Female: 601 (50.8%) Missing/Not Specified: 3 (0.3%)	Hispanic 64 (5.4%) Non-Hispanic White 271 (22.9%) Non-Hispanic Black 19 (1.6%) Non-Hispanic AI/AN 1 (0.1%) Non-Hispanic Asian 8 (0.7%) Non-Hispanic NHOPI 0 (0.0%) Non-Hispanic Multi-Racial 36 (3.0%) Non-Hispanic Other 4 (0.3%) Missing/Not Reported 781 (66.0%)	
Simons Searchlight	Version 3.1	571	7/13/22 − 10/19/22	106.5 (55.9)	Male: 279 (48.9%) Female: 218 (38.2%) Missing/Not Specified: 73 (13.0%)	Hispanic 22 (3.9%) Non-Hispanic White 78 (13.7%) White (missing ethnicity) 105 (18.4%) Black (missing ethnicity) 2 (0.4%) Non-Hispanic Asian 2 (0.4%) Asian (missing ethnicity) 4 (0.7%) Non-Hispanic Multi-Racial 10 (1.8%) Multi-Racial (missing ethnicity) 11 (1.9%) Other 3 (0.5%) Missing/Not Reported 334 (58.5%)	Simons Searchlight is an initiative to better understand genetic neuro-developmental conditions and their associations with autism.

### Statistical analysis

Across all datasets, item responses were examined for correlations with chronological age and for floor and ceiling effects, both in aggregate and within groups based on caregiver-reported NDD diagnosis, age, and language. Items with strong floor or ceiling effects were tagged for revision or elimination. Because of our developmental framework, we expected the endorsement of each item (representing a skill or capacity) to increase with age, especially among children without NDD diagnoses. When rate of item endorsement was uncorrelated with or showed variable relationships with age (e.g., negatively correlated at older ages), it was eliminated.

Within each version of the candidate item bank, we conducted dimensionality analysis. Consistent with best practices in exploratory factor analysis [32], we used multiple criteria for determining the optimal number of factors to extract. These included the ratio of first-to-second eigenvalues, examination of the scree plot, and use of Velicer’s minimal average partial [33]. If multidimensional structures were suggested, we also examined the interpretability of rotated pattern of factor loadings, using both oblique quartimin and biquartimin rotations [34, 35]. 

Because the overlapping items from Versions 3.0 and 3.1 were nearly or perfectly identical, data from waves using these versions were aggregated and submitted to modern measurement models: dimensionality analyses, item response theory (IRT) calibration, and differential item functioning (DIF) analyses. IRT modeling used the unidimensional or multidimensional graded response model [36, 37]. DIF analyses were performed within a multiple group framework, with separate analyses for age, sex assigned at birth, and language level. For DIF analyses, a fully constrained IRT model was fit to both groups with freely estimated mean and variances in the focal group. Then cross-group constraints for each item were systematically released for nested model comparisons [38]. In addition to evaluating the [39] statistical significance of the DIF analyses (with adjusted p-values using the false discovery rate [39]), we also evaluated the practical significance through an impact analysis [40–42]. Consistent with best practice recommendations [43, 44], we used the weighted Area Between the Curves (wABC) [45], the Signed Item Difference in the Sample (SIDS) and the Signed Test Differences in the Sample (STDS), and finally the Expected Score Standardized Difference (ESSD) and the Expected Test Score Standardized Difference (ETSSD) [44]. The wABC can be interpreted as potentially concerning when greater than 0.35, and the ESSD and ETSSD can be interpreted similarly to Cohen’s *d*.

## Results

### Focus groups and cognitive debriefing interviews

In addition to specific wording suggestions, several themes emerged from parent/caregiver feedback. First, the meaning of the word “currently” in the question stem was unclear to parents. We therefore modified the question stem to refer to a specific recall window of two weeks. Second, parents described that a given behavior might occur in some contexts but not in others. They recommended that each item specify a context, such as where or with whom the use of a skill should be considered. Third, caregivers of younger children or those with more significant developmental delays or intellectual disability often mentioned that the skills surveyed were too advanced for their children. This indicated insufficient item coverage for social communication skills relevant to children at lower developmental levels. Fourth, the caregivers identified questions that required inference of internal state or motivation of their children. This is less useful for caregiver-reported outcomes, which should pertain only to observable behaviors. Fifth, the caregivers reported having limited opportunities to observe the skills referenced by some items, which was likely exacerbated by COVID-19 social distancing practices. Caregivers felt that they were unable to validly report about their children’s social communication behaviors outside of the home or with peers (besides siblings). Although we did not revise the item bank to specifically accommodate the unusual situation created by the COVID-19 pandemic, this feedback did highlight the need to focus on skills that would be observable by a caregiver regardless of whether they regularly spent time with the child outside of the home. As described below, each of these themes was carefully considered in revisions to the item bank.

### Field testing

Table 1 provides information on each stage of data collection, including participant ages and sample size. Additional child and informant demographics are provided in Supplementary Table S1. While the market research firm samples were unselected for developmental concerns [46, 47], about 20% of parents in each wave of data collection indicated that their child had been diagnosed with one or more developmental delays or NDDs, consistent with population-based estimates [46, 47].

*Feasibility.* Although very few items were excluded due to floor effects, ceiling effects were common in early versions of the item pool, especially for older, more verbal children. Social communication abilities develop rapidly, so including older individuals unselected for developmental concerns resulted in minimal variance on several items. Additional items were eliminated due to unexpected relationships with chronological age.

*Dimensionality.* Preliminary dimensionality analyses were conducted after each round of data collection. In Version 1.0 of the item bank, dimensionality was examined separately for items that were applicable regardless of language level (general set) versus those only applicable to individuals with at least phrase speech (advanced set). For both item sets, the eigenvalue ratio suggested a single general factor, and Velicer’s minimal average partial [33] suggested two factors. Both two-factor solutions had a primary factor representing overall social communication, but the content of the secondary factor varied. For the general set, the secondary factor concerned whether a child was able to effectively make requests (e.g., “If your child is hungry, does he/she let you know?”), and for the advanced set it concerned whether the child was able to appropriately modulate use of certain behaviors (e.g., “Does your child stop talking about something when others don’t want to talk about that?”). However, all but one item from the advanced item set (about appropriately using text message to communicate) primarily loaded on the overall social communication factor. As a result, we determined that the outlying item did not fit within our conceptual framework and eliminated it.

Dimensionality analyses were conducted again for the Version 2.0 bank. Using all items, the eigenvalue ratio suggested one primary factor, but the scree plot suggested that a second factor might explain meaningful response variability. Exploratory factor models suggested that while one factor reflecting general social communication skills included almost all items, the second factor reflected the few negatively worded deficit-focused items that we initially included on our ability-focused measure (e.g., the reverse-coded item “Say something that might make someone feel bad or embarrassed”). Thus, these negatively worded items were removed or re-written to be ability-focused for Version 3.0 and subsequent iterations.

Dimensionality analyses of aggregated data from Versions 3.0 and 3.1 also supported one primary social communication factor. The eigenvalue ratio suggested one factor, scree plot suggested three factors, and Velicer’s minimal average partial suggested eight factors. These multidimensional structures were interpreted as one primary social communication factor, with additional factors capturing residual item covariance for similarly worded items (e.g., item stems starting with “look at…”), items sharing a social context, or items of approximately similar difficulty. Items that were only weakly associated with the primary social communication dimension were eliminated.

After these multi-staged analyses, a measure with 184 items fit a one-primary-factor social communication model with multiple secondary factors that explain residual variance. We used the accumulated results to place the items into mutually exclusive developmental categories, which would only be administered to individuals based on whether they spoke in phrases or better. This was done by comparing item endorsement rates to the responses on the language routing items (described above). From the full item set, 62 were labeled appropriate for both language levels, 79 were labeled as appropriate only for more verbal individuals, and 43 were labeled as appropriate only for less verbal individuals. This language-stratified bifactor structure with one primary social communication factor and three nuisance factors was moved to the next phase of confirmatory IRT modeling. Unexpectedly, despite multiple attempts to group items into different “types” of social communication behaviors (e.g., based on form or purpose of initiation or response), models incorporating multiple social communication factors or attributes did not substantially improve the statistical model fit for these data. Thus, we hypothesize that social communication may mirror other domains of development where early-emerging skills that appear to represent different categories develop nearly simultaneously (e.g., young children begin using verbs shortly after first nouns are acquired) [48]. 

**Fig. 1:**
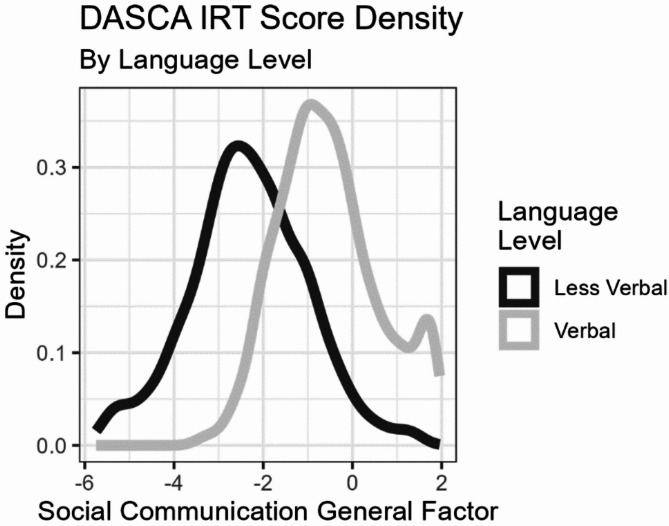
DASCA Score Density by Language Level

*Item Response Theory Modeling.* Following the dimensionality assessment, item-level data from Version 3.1 (together with data from the overlapping items from Version 3.0) were submitted for IRT modeling [36, 37]. A two-group structure reflecting the language grouping was specified. The more verbal group defined the scale distribution (mean = 0.0, SD = 1.0 for all factors), so the factor score distributions were estimated for the less verbal group. As expected under our developmental framework, the less verbal group had lower social communication ability, with a theta score distribution of mean=-2.36 SD = 1.38. All parameters for the final IRT model are provided in Supplementary Table 2, and the distribution of IRT-based scores on the general factor are shown in Fig. 1.

*Differential Item Functioning.* DIF on the substantive social communication factor was examined by language level, chronological age, and sex assigned at birth. For DIF by language level, we only tested the 62 items appropriate for both language groups; for chronological age, we examined items within language level and dichotomized age at 4 years for the less verbal group or at 9 years for the more verbal group (the approximate medians in each group). For sex assigned at birth, all items were considered together, marginalizing across language level subsets.

By language level, 13 of the 62 items were flagged as potentially exhibiting statistically significant DIF. None of these had ΔBIC > 3, though, which suggests that they are statistically near-equivalent models. To evaluate the impact of these items, we fitted a constrained model where the other items served as an anchor and item- and test-level impact analyses were conducted. No items had wABC > 0.35; in the observed-score metric using the SIDS, one item exhibited potentially impactful DIF with a SIDS = 0.31; in the standardized item-level difference using the ESSD, no items had a medium effect (|ESSD|≥0.50), but seven had small effects (|ESSD|≥0.20). At the test level, these differences largely canceled out, with an observed score difference of less than 1 point overall (STDS = 0.61) and minimal standardized score difference (ETSSD = 0.01).

For chronological age in the less verbal group, 21 of the 105 items (which included the general item set and those only appropriate for the less verbal group) were flagged as potentially exhibiting statistically significant DIF. Of these, five had ΔBIC > 3. Several of these differences were potentially large enough to impact scores at the item level: 10 items had wABC > 0.35, seven had |SIDS|>0.50, and 12 had |ESSD|≥0.50. At the test level, this would result in an observed score difference of STDS = 5.9 points, with higher ability in the older age group. However, this corresponds to a standardized effect of ETSSD = 0.08, which is small. Together, these results suggest that some items exhibit age-related DIF but in the context of the entire item bank, the effect is small.

A similar approach was used to investigate age-related DIF for the more verbal group, with 63 of the 141 items flagged as exhibiting potentially statistically significant DIF. Only 14 had ΔBIC > 3. Again, several of the item-level indices suggested a potential for score-level differences: nine items had wABC > 0.35; two had |SIDS|>0.50; and 14 items had |ESSD|≥0.50. However, at the test level, the observed score difference was less than one point (STDS = 0.63), which on the standardized metric was negligible (ETSSD = 0.007). Lastly, for sex assigned at birth, none of the 184 items was flagged as exhibiting potentially problematic DIF after correction. Therefore, we had no items to submit to an impact analysis.

In summary, DIF analyses suggested some instances of statistically significant item-level DIF. However, follow-up impact analyses showed that the practical significance at the test level resulted in minimal to no differences. There are some individual items with practical impact for age-related DIF, but there are no practical ramifications at the test-level interpretation for any comparison. Thus, while item-level DIF may occur, it is relatively rare and unimpactful overall [40]. 

## Discussion

Psychology lacks quantitative measures of social communication that are sensitive to changes within individuals over time. The measurement of social communication has overwhelmingly focused on impairments relevant to the diagnosis of autism, rather than skill acquisition across development or in response to intervention. Using best practices from measurement science and test development—including both qualitative and quantitative standards [23, 30, 42]—we developed the DASCA, an ability-focused measure of social communication.

We defined social communication as “the appropriate use and modulation of verbal and nonverbal behaviors during interactions with others” (p.555) [1]. Our initial conceptual framework emphasized using and modulating basic social communication ability, cognitive and language abilities, attention, social cognition, emotion regulation, and behavioral inhibition within an evolving social milieu [8]. Throughout our initial measure development efforts, we modified this conceptual framework to reflect the qualitative results from concept elicitation, focus groups, the cognitive debriefing interviews, and other feedback from professional and family/caregiver community partners. The largest modification to our conceptual framework was differentiating between theoretical aspects of social communication versus aspects that can be feasibly and reliably measured via caregiver report. For example, findings from our mixed methods approach continue to support social cognition and emotion regulation as important components of social communication, particularly starting in middle to later childhood. Still, measurement of these skills requires the parent or caregiver to infer internal states and motivations. Further, our attempts to quantify these components highlighted the role of individual or personality differences—particularly at more mature developmental levels—that were not the intended focus of the measure. Measurement limitations, then, place constraints on quantifying all aspects of our conceptual model. Therefore, for the DASCA measure, we chose to focus on observable social communication behaviors relevant to the early developmental period, when most social interactions occur with or in the presence of primary caregivers.

Previous analyses of items from autism symptom measures (such as the ADOS-2) to support the existence of multiple types of social communication difficulties [9, 10, 49]. However, after each revision of the DASCA item pool, the tests of dimensionality confirmed the predominance of one general social communication factor, where components such as initiation, response, and maintenance of social interaction are integrally related. Therefore, while there appears to be subtypes of social communication *impairment*, our qualitative and quantitative findings suggest that social communication ability is more difficult to parse into distinct components, at least as measured by caregiver-report.

In Versions 3.0 and 3.1, we were able to test for potential DIF on the general social communication factor. Neither sex assigned at birth nor language level was associated with practically important DIF effects at the test level. Some potentially impactful item-level age-related DIF was observed within the language level subsets. Still, at the test-level, these effects were small and overpowered by the items that did not exhibit DIF. This would be consequential for administration via computerized adaptive testing if only the DIF items are administered, and so we plan to implement item-administration rules to prevent over-utilization of these items within a single adaptive administration of the DASCA. Taken together, the DIF results suggest that our model and scoring procedures for the DASCA item bank are robust against construct-irrelevant DIF due to language level, chronological age, or sex assigned at birth, supporting its validity as a measure of social communication ability across groups, particularly when examining overall scores rather than individual items.

As expected, our preliminary analyses of DASCA items indicated that while some items are appropriate regardless of an individual’s language level, the suitability of other items depends on language level. As a result, we calibrated the DASCA item bank using a bifactor model with the multidimensional graded response model [36, 37]. 

Using these IRT calibrations, we developed a computerized adaptive test version of the DASCA. Thus, items from the DASCA item bank will be administered as part of the computer adaptive assessment, rather than as long forms with large numbers of items. In this way, a respondent does not need to complete all of the items to get a meaningful score when using modern measurement theory. Based on the language routing items, the adaptive version will administer items targeted to language level, with exposure control and item selection rules to make it appropriate for high-frequency assessment without over-utilizing the same items in the child’s range of ability. Field testing and psychometric evaluation of the adaptive version of the DASCA are ongoing.

## Limitations

Although we conducted exploratory dimensionality assessments for each version of the DASCA candidate item bank, for the confirmatory IRT modeling, we used the same dataset (Version 3.1 of the item bank) as was used for dimensionality assessments. The overall sample was large, but the planned missing design [31] and our intention to examine DIF by multiple subgroups meant that we could not follow the best practice of separate samples for exploratory and confirmatory phases of modeling [20, 32]. The dimensionality proposed herein should be validated in an independent dataset.

A second limitation is that much of the data were collected during the COVID-19 pandemic. Social behaviors—even for highly adaptable individuals—were constrained during this period, making it difficult for some caregivers to report on “typical” social interactions for the child whom they were rating. It is possible that post-COVID social mores may also have changed, which, on the one hand, strengthens the argument for having caregivers report only on social interactions they are a part of, but on the other hand, raises additional questions about developmentally-normative modulation of social behavior in peer- and community-settings. Regardless, because caregivers are not always able to consistently observe or validly report on social behavior with peers, this is an important area for future measure development.

## Conclusions

The DASCA item bank represents a significant advance in measuring caregiver-reported social communication in children with NDDs. Unlike previous measures, it focuses on the presence of developmentally expected social communication skills rather than lack thereof. As a result, it offers an advantage over existing measures in both the breadth and depth of abilities captured, potentially allowing for a more responsive measurement of social communication. This is especially relevant for clinical trials or intervention programs aimed at improving social communication in NDD populations. Importantly, the careful development process of the DASCA allows for confidence in its use beyond very early childhood, a period during which core components of social communication ability typically emerge. This is an important advantage for NDD populations, as even basic social communication skills may not fully develop until later. Thus, DASCA provides a vital step forward in quantifying social communication ability.

## Supplementary Information

Below is the link to the electronic supplementary material.

Supplementary Material 1

## Data Availability

Data for this study were collected in multiple waves. For those collected through the Opinions4Good vendor, data are available by request of the corresponding author. Data included in the current study which were collected through Simons SPARK or Searchlight are available on the SPARK Research Match registry hosted by Simons Foundation. Interested researchers can receive approval and obtain the SPARK population dataset described in this study by applying at https://base.sfari.org.

## Abbreviations

ΔBIC: Change in Bayesian information criterion
DASCA: Developmental Assessment of Social Communication Ability
DIF: Differential item functioning
ESSD: Expected Score Standardized Difference
ETSSD: Expected Test Score Standardized Difference
IRT: Item response theory
NDD: Neurodevelopmental disorder
Op4G: Opinions4Good
SD: Standard Deviation
SIDS: Signed Item Difference in the Sample
STDS: Signed Test Differences in the Sample
VABS: Vineland Adaptive Behavior Scales
wABC: weighted Area Between the Curves

## Footnotes

1 Note: at the time of manuscript submission, the market research firm and a close business affiliate are under investigation for utilizing “ant” respondents. Most of the affected studies had been transitioned to the business affiliate, with which we did not engage. While we have no direct information that our data were affected, we also cannot conclusively attest that all participants were who they said they were.
