# Supplementary material for: The Developmental Assessment of Social Communication Ability (DASCA): initial creation and psychometric description
Source: Mol Autism. 2025 Oct 16;16:52. doi: 10.1186/s13229-025-00683-z (PMC12529783; doi:10.1186/s13229-025-00683-z)
Supplement: Supplementary file 1 — Supplementary Material 1 [file 13229_2025_683_MOESM1_ESM.pdf]

DASCA Supplementary Online Materials

Supplementary Table 1: Participant Demographics by Dataset

		Op4G Wave 1		Op4gG Wave 2		Op4G Wave 3		SPARK Probands		SPARK Siblings		Searchlight		Overall	
**Demographic**	**Characteristic**	**N**	**%**	**N**	**%**	**N**	**%**	**N**	**%**	**N**	**%**	**N**	**%**	**N**	**%**
**Parental Education**	**Some High School or Less**	58	5.8	11	1.1	18	1.6	24	2.2	1	0.1	6	1.1	118	2
	**High School Graduate or GED**	150	15	265	26.5	300	26.1	153	14.1	9	0.8	42	7.4	919	15.3
	**Some College, No Degree**	167	16.7	42	4.2	†	†	194	17.8	16	1.4	88	15.4	507	8.5
	**Associates Degree or Trade School**	131	13.1	369	36.9	†	†	216	19.9	20	1.7	42	7.4	778	13
	**Bachelor's Degree (BA/BS)**	315	31.5	72	7.2	346	30.1	270	24.8	18	1.5	168	29.4	1189	19.8
	**Advanced Degree (Master's, Doctorate, or Professional)**	179	17.9	241	24.1	486	42.3	220	20.2	19	1.6	149	26.1	1294	21.6
	**Missing**	0	0	0	0	0	0	10	0.9	1101	93	76	13.3	1187	19.8
**Child Sex Assigned at Birth**	**Male**	569	56.9	514	51.4	576	50.1	835	76.8	580	49	279	48.9	3353	56
	**Female**	431	43.1	486	48.6	574	49.9	246	22.6	601	50.8	218	38.2	2556	42.7
	**Missing**	0	0	0	0	0	0	6	0.6	3	0.3	74	13	83	1.4
**Ethnicity and Race**	**Hispanic**	238	23.8	134	13.4	191	16.6	191	17.6	64	5.4	22	3.9	840	14
	**Non-Hispanic White**	589	58.9	676	67.6	733	63.7	722	66.4	271	22.9	78	13.7	3069	51.2
	**Non-Hispanic Black**	77	7.7	67	6.7	80	7	41	3.8	19	1.6	0	0	284	4.7
	**Non-Hispanic American Indian or Alaskan Native**	6	0.6	3	0.3	16	1.4	1	0.1	1	0.1	0	0	27	0.5

	**Non-Hispanic Asian**	40	4	52	5.2	62	5.4	28	2.6	8	0.7	2	0.4	192	3.2
	**Non-Hispanic Native Hawaiian or Other Pacific Islander**	3	0.3	1	0.1	7	0.6	1	0.1	0	0	0	0	12	0.2
	**Non-Hispanic Multi-Racial**	47	4.7	67	6.7	61	5.3	84	7.7	36	3	10	1.8	305	5.1
	**Non-Hispanic Other**	†	†	†	†	†	†	12	1.1	4	0.3	0	0	16	0.3
	**White (Ethnicity Not Specified)**	0	0	0	0	0	0	0	0	0	0	105	18.4	105	1.8
	**Black (Ethnicity Not Specified)**	0	0	0	0	0	0	0	0	0	0	2	0.4	2	0
	**Asian (Ethnicity Not Specified)**	0	0	0	0	0	0	0	0	0	0	4	0.7	4	0.1
	**Multi-Racial (Ethnicity Not Specified)**	0	0	0	0	0	0	0	0	0	0	11	1.9	11	0.2
	**Other (Ethnicity Not Specified)**	0	0	0	0	0	0	0	0	0	0	3	0.5	3	0.1
	**Missing**	0	0	0	0	0	0	7	0.6	781	66	334	58.5	1122	18.7
**Autism Spectrum Disorder**	**No**	924	92.4	919	91.9	1072	93.2	0	0	599	50.6	283	49.6	3797	63.4
	**Yes**	76	7.6	81	8.1	78	6.8	1087	100	151	12.8	260	45.5	1733	28.9
	**Missing**	0	0	0	0	0	0	0	0	434	36.7	28	4.9	462	7.7
**ADHD**	**No**	897	89.7	889	88.9	1052	91.5	†	†	626	52.9	382	66.9	3846	64.2
	**Yes**	103	10.3	111	11.1	98	8.5	†	†	124	10.5	161	28.2	597	10
	**Missing**	0	0	0	0	0	0	†	†	434	36.7	28	4.9	1549	25.9
**Language Disorder**	**No**	938	93.8	950	95	1024	89	†	†	734	62	240	42	3886	64.9

	**Yes**	62	6.2	50	5	126	11	†	†	16	1.4	303	53.1	557	9.3
	**Missing**	0	0	0	0	0	0	†	†	434	36.7	28	4.9	1549	25.9
**Intellectual Disability or Global Developmental Delay**	**No**	†	†	†	†	1052	91.5	†	†	736	62.2	102	17.9	1890	31.5
	**Yes**	†	†	†	†	98	8.5	†	†	14	1.2	441	77.2	553	9.2
	**Missing**	†	†	†	†	0	0	†	†	434	36.7	28	4.9	3549	59.2
**Age in Months**	**Mean (SD), Range**	51.7 (36.2)	2-119	77.2 (57.4)	6-216	54.4 (50.1)	12-216	112.2 (47.3)	26-214	130.3 (49.7)	29-215	104.9 (55.1)	8-216	89.0 (57.1)	2-216

† = Question not asked for this round of data collection

Supplementary Table 2: Item Response Theory Modeling Item Parameters

Item ID	Item Group	Overall Social Comm.	a2	a3	a4	d1	d2	d3
v3d099	Both	2.521	0	0.795	0	3.824	1.697	-0.357
v3d045	Both	2.41	0	1.312	0	3.673	1.371	-1.139
v3d143	Both	2.355	0	0	0	4.191	2.433	0.568
v3d257	Both	2.223	0	0.413	0	3.898	2.287	0.418
v3d102	Both	2.151	0	2.009	0	2.393	0.116	-2.209
v3d134	Both	2.064	0	0	0	3.486	1.952	0.25
v3d294	Both	2.046	0	0	0	4.625	2.707	0.967
v3d084	Both	2.039	0	0	0	4.379	2.736	0.947
v3d077	Both	2.02	0	0.045	0	4.681	2.809	0.905
v3d081	Both	1.975	0	0	0	5.032	3.272	1.507
v3d031	Both	1.955	0	0	0	5.502	3.347	1.304
v3d251	Both	1.936	0	0.337	0	4.16	2.38	0.463
v3d186	Both	1.934	0	0	0	3.776	2.201	0.384
v3d037	Both	1.916	0	0	0	5.013	3.139	1.375
v3d018	Both	1.912	0.663	0	0	6.695	4.708	2.571
v3d104	Both	1.909	0	0	0	3.625	1.782	-0.095
v3d082	Both	1.9	0	0	0	4.117	2.216	0.165
v3d187	Both	1.894	0	0.397	0	4.199	2.713	1.064
v3d086	Both	1.881	0	0	0	4.071	2.604	0.956
v3d226	Both	1.88	0	0	0	6.359	4.044	2.179
v3d216	Both	1.88	0.498	0	0	5.364	3.689	1.938
v3d100	Both	1.879	0	0.588	0	3.76	1.876	0.075
v3d283	Both	1.876	0	0	0	4.573	2.876	1.199
v3d063	Both	1.873	0	0	0	5.82	3.516	1.609
v3d078	Both	1.871	0	1.665	0	3.188	1.184	-0.787
v3d189	Both	1.835	0	0	0	5.139	2.975	0.9
v3d110	Both	1.751	0	0	0	4.957	2.757	0.904
v3d203	Both	1.742	0	1.113	0	3.053	0.941	-1.107
v3d154	Both	1.722	0	0	0	3.972	1.81	-0.268
v3d142	Both	1.713	0	0.466	0	3.658	1.857	-0.029
v3d310	Both	1.697	0	0.812	0	3.431	1.373	-0.582
v3d127	Both	1.668	0	0	0	4.537	2.673	1.146
v3d311	Both	1.63	0	0.989	0	2.968	0.905	-0.947
v3d237	Both	1.625	0	0	0	5.108	3.167	1.445
v3d073	Both	1.625	0.317	0	0	5.339	3.494	1.951
v3d228	Both	1.594	0	0	0	5.352	3.214	1.437
v3d282	Both	1.567	0	0	0	5.526	3.52	1.635
v3d062	Both	1.559	0.871	0	0	6.365	3.916	1.654
v3d286	Both	1.555	0	0	0	4.497	2.576	1.171

Item ID	Item Group	Overall Social Comm.	a2	a3	a4	d1	d2	d3
v3d173	Both	1.537	0	0	0	6.345	3.556	1.894
v3d015	Both	1.525	0	0	0	3.966	2.344	0.854
v3d025	Both	1.516	0	0	0	4.316	2.391	0.628
v3d295	Both	1.511	0	0	0	3.371	2.012	0.526
v3d185	Both	1.497	0	0	0	5.084	2.819	1.256
v3d262	Both	1.468	0	0	0	3.51	1.605	0.108
v3d083	Both	1.454	0.534	0	0	5.508	3.652	2.174
v3d287	Both	1.438	1.407	0	0	6.92	4.828	2.945
v3d165	Both	1.435	0	0	0	3.392	2.097	0.804
v3d253	Both	1.415	0	0	0	3.531	1.709	0.006
v3d319	Both	1.408	0	0.953	0	3.078	1.189	-0.282
v3d292	Both	1.396	1.343	0	0	6.072	4.019	2.199
v3d056	Both	1.362	0	0	0	3.825	2.252	0.732
v3d091	Both	1.345	0	0	0	3.807	1.979	0.623
v3d312	Both	1.307	0	1.054	0	3.417	1.425	-0.255
v3d175	Both	1.301	0	0	0	3.979	2.538	1.214
v3d235	Both	1.276	0	0	0	4.103	2.781	1.303
v3d107	Both	1.172	0	0.913	0	2.911	1.409	-0.086
v3d123	Both	1.163	0	0	0	3.809	2.082	0.7
v3d272	Both	1.142	0	0	0	3.522	1.207	-0.671
v3d022	Both	1.142	1.338	0	0	5.781	4.051	2.251
v3d224	Both	1.124	0	0	0	3.543	2.035	0.767
v3d184	Both	1.081	0	0	0	2.92	1.599	0.268
v3d148	More Verbal	3.415	0	0.965	0	6.379	3.072	0.174
v3d301	More Verbal	3.283	0	0	2.015	6.17	3.785	1.487
v3d149	More Verbal	3.014	0	0	1.744	5.804	3.451	1.214
v3d094	More Verbal	2.791	0	0	0.82	5.957	3.974	1.711
v3d248	More Verbal	2.748	0	0	0	6.422	3.584	1.379
v3d255	More Verbal	2.693	0	0	1.157	5.726	3.621	1.489
v3d210	More Verbal	2.686	0	1.731	0	4.3	1.491	-0.447
v3d069	More Verbal	2.606	0	0	0	5.703	2.957	0.56
v3d158	More Verbal	2.554	0	1.771	0	3.694	1.44	-1.071
v3d093	More Verbal	2.46	0	0	1.743	5.063	2.912	0.998
v3d092	More Verbal	2.436	0	0	0	4.721	2.9	0.813
v3d032	More Verbal	2.43	0	0	1.091	5.604	3.436	1.726
v3d305	More Verbal	2.417	0	0	1.347	4.093	2.614	0.495
v3d146	More Verbal	2.394	0	0	1.269	4.404	2.587	0.646
v3d190	More Verbal	2.389	0	0	0	4.912	2.723	0.703
v3d302	More Verbal	2.363	0	0	0.671	6.132	3.731	1.714
v3d013	More Verbal	2.362	0	0	0	7.473	4.409	2.106

Item ID	Item Group	Overall Social Comm.	a2	a3	a4	d1	d2	d3
v3d298	More Verbal	2.348	0	0	1.178	5.767	3.28	1.166
v3d138	More Verbal	2.344	0	0	0	4.706	2.808	0.862
v3d205	More Verbal	2.329	0	0	0	4.473	2.673	0.938
v3d095	More Verbal	2.312	0	0	0	4.788	2.695	0.9
v3d299	More Verbal	2.301	0	0	0.601	4.929	3.211	1.531
v3d306	More Verbal	2.293	0	0	0.861	3.623	1.956	0.176
v3d039	More Verbal	2.292	0	0	0	4.216	2.315	0.157
v3d144	More Verbal	2.28	0	0	0.571	4.095	2.352	0.504
v3d199	More Verbal	2.255	0	0.309	0	4.896	3.026	1.207
v3d137	More Verbal	2.254	0	0	0	4.713	3.239	1.607
v3d159	More Verbal	2.254	0	2.402	0	3.177	0.898	-1.328
v3d247	More Verbal	2.251	0	0	0.697	3.671	2.135	0.537
v3d300	More Verbal	2.211	0	0	1.026	5.75	3.994	2.022
v3d089	More Verbal	2.198	0	0	0.188	5.516	3.398	1.434
v3d061	More Verbal	2.197	0.827	0	0	6.278	4.284	2.373
v3d101	More Verbal	2.188	0	1.65	0	2.547	0.638	-1.3
v3d076	More Verbal	2.174	0	0	0.979	5.161	3.551	1.713
v3d157	More Verbal	2.171	0	1.39	0	3.161	1.565	-0.01
v3d196	More Verbal	2.151	0	0	0	3.967	2.33	0.645
v3d198	More Verbal	2.146	0	0	0	3.668	2.178	0.354
v3d085	More Verbal	2.142	0	0	0	4.817	3.092	0.991
v3d192	More Verbal	2.142	0	0	0	5.078	3.144	1.586
v3d141	More Verbal	2.134	0	0	0.698	5.455	3.623	1.694
v3d140	More Verbal	2.1	0	0	0	4.841	3.566	1.867
v3d035	More Verbal	2.086	0	0	0	5.213	2.99	1.118
v3d263	More Verbal	2.067	0	1.592	0	3.411	0.995	-1.224
v3d245	More Verbal	2.053	0	0	0	6.149	3.542	1.231
v3d116	More Verbal	2.036	0	0	0	5.698	2.717	0.39
v3d105	More Verbal	2.021	0	1.77	0	1.997	0.392	-1.643
v3d139	More Verbal	2.014	0	0	0	4.944	2.801	1.07
v3d147	More Verbal	1.942	0	0.511	0	4.202	2.167	0.465
v3d145	More Verbal	1.941	0	0	0.739	3.625	1.925	0.302
v3d258	More Verbal	1.939	0	0	0	3.721	1.857	-0.037
v3d252	More Verbal	1.936	0	1.103	0	3.649	1.799	-0.228
v3d096	More Verbal	1.92	0	0	0	3.89	2.54	0.823
v3d234	More Verbal	1.893	0	0	0	6.081	3.696	1.815
v3d193	More Verbal	1.889	0	0	0	6.247	4.352	2.374
v3d250	More Verbal	1.88	0	0	0	5.885	3.337	1.302
v3d029	More Verbal	1.856	0	0.34	0	3.828	2.57	0.924
v3d038	More Verbal	1.848	0	0	0	4.491	2.55	0.693

Item ID	Item Group	Overall Social Comm.	a2	a3	a4	d1	d2	d3
v3d246	More Verbal	1.844	0.612	0	0	5.634	3.954	2.18
v3d316	More Verbal	1.836	0	0	1.018	3.611	2.205	0.544
v3d114	More Verbal	1.835	0	0	0	4.314	1.871	0.012
v3d103	More Verbal	1.826	0	1.028	0	2.442	0.892	-0.677
v3d204	More Verbal	1.823	0	0	0	5.385	3.384	1.53
v3d254	More Verbal	1.818	0	0	0	3.685	2.097	0.379
v3d001	More Verbal	1.815	0	0	0	5.716	3.428	1.587
v3d033	More Verbal	1.792	0	0	0	4.533	2.673	0.933
v3d229	More Verbal	1.788	0	0	0	4.659	2.494	0.779
v3d048	More Verbal	1.748	0	0	0	2.665	1.186	-0.321
v3d060	More Verbal	1.704	0	0	0	5.028	2.623	0.749
v3d046	More Verbal	1.64	0	0	0	3.008	1.508	-0.125
v3d133	More Verbal	1.64	0	0	0	4.036	2.461	0.686
v3d268	More Verbal	1.585	0	0.905	0	1.179	0.112	-0.959
v3d314	More Verbal	1.567	0	0	0.768	3.702	2.308	0.947
v3d195	More Verbal	1.541	0	0	0	3.16	1.22	-0.182
v3d053	More Verbal	1.527	0	1.1	0	1.795	0.179	-1.383
v3d179	More Verbal	1.49	0	0	0	4.994	3.076	1.25
v3d315	More Verbal	1.483	0	0	0.537	2.944	1.497	0.4
v3d207	More Verbal	1.445	0	0	0	2.791	1.15	-0.244
v3d201	More Verbal	1.414	0	0	0	2.411	0.858	-0.605
v3d320	More Verbal	1.384	0	1.168	0	1.392	0.031	-1.598
v3d188	Less Verbal	2.435	0	0	0	5.109	3.848	1.825
v3d126	Less Verbal	2.224	0.652	0	0	6.866	5.21	3.582
v3d128	Less Verbal	2.174	-0.44	0	0	4.705	3.217	1.792
v3d021	Less Verbal	1.987	0	0	0	4.741	2.992	1.216
v3d071	Less Verbal	1.939	0.162	0	0	5.14	3.737	2.188
v3d180	Less Verbal	1.882	0	0	0	4.284	2.824	1.098
v3d059	Less Verbal	1.792	0.433	0	0	6.134	4.103	2.458
v3d136	Less Verbal	1.782	0.382	0	0	5.716	3.941	1.896
v3d008	Less Verbal	1.753	0	0	0	5.71	3.309	1.15
v3d168	Less Verbal	1.657	0	0	0	4.291	2.706	1.209
v3d222	Less Verbal	1.637	0	0	0	5.112	2.551	0.821
v3d122	Less Verbal	1.633	1.09	0	0	6.133	4.38	2.939
v3d009	Less Verbal	1.612	0	0	0	3.316	1.869	0.24
v3d167	Less Verbal	1.6	0	0	0	5.97	3.917	2.138
v3d019	Less Verbal	1.555	0.456	0	0	5.652	3.812	2.241
v3d233	Less Verbal	1.539	0	0	0	4.052	2.624	1.04
v3d065	Less Verbal	1.505	0	0	0	5.392	3.325	1.663
v3d273	Less Verbal	1.489	1.258	0	0	7.888	4.944	2.892

Item ID	Item Group	Overall Social Comm.	a2	a3	a4	d1	d2	d3
v3d285	Less Verbal	1.481	1.221	0	0	6.164	4.759	3.378
v3d002	Less Verbal	1.465	0	0	0	4.288	2.857	1.307
v3d068	Less Verbal	1.403	1.817	0	0	7.698	5.206	3.052
v3d208	Less Verbal	1.395	0	0	0	3.682	2.521	1.39
v3d231	Less Verbal	1.363	0.512	0	0	5.29	3.507	2.136
v3d304	Less Verbal	1.362	0	0	0	3.073	2	0.612
v3d066	Less Verbal	1.347	0.667	0	0	4.895	3.356	1.957
v3d215	Less Verbal	1.289	0	0	0	3.902	2.503	1.434
v3d182	Less Verbal	1.269	1.633	0	0	7.295	5.495	3.774
v3d166	Less Verbal	1.256	0	0	0	5.403	2.986	1.558
v3d024	Less Verbal	1.227	0	0	0	4.08	2.411	1
v3d111	Less Verbal	1.224	0.098	0	0	4.196	2.41	1.344
v3d171	Less Verbal	1.19	2.458	0	0	7.27	5.462	3.615
v3d218	Less Verbal	1.182	1.559	0	0	6.821	4.804	2.867
v3d176	Less Verbal	1.121	1.178	0	0	5.012	3.641	2.193
v3d012	Less Verbal	1.108	0	0	0	4.433	2.991	1.804
v3d117	Less Verbal	1.069	2.234	0	0	6.481	4.901	3.1
v3d275	Less Verbal	1.03	1.229	0	0	5.512	4.094	2.6
v3d011	Less Verbal	1.029	2.769	0	0	8.829	6.349	4.526
v3d064	Less Verbal	0.978	1.774	0	0	5.98	4.485	2.68
v3d113	Less Verbal	0.94	1.042	0	0	6.065	4.197	2.48
v3d164	Less Verbal	0.927	0	0	0	3.724	2.652	1.849
v3d125	Less Verbal	0.896	1.04	0	0	4.797	3.396	2.096
v3d221	Less Verbal	0.89	0.982	0	0	4.6	3.172	2.112
v3d223	Less Verbal	0.77	1.414	0	0	4.528	3.468	2.271

Note: The first factor is the general factor reflecting overall social communication. We are not creating a name for factors a2, a3, and a4 insofar as they are nuisance factors representing similarly-worded items, items that share a similar context, or that otherwise reflect less relevant content.
